# Protein annotation as term categorization in the gene ontology using word proximity networks

**DOI:** 10.1186/1471-2105-6-S1-S20

**Published:** 2005-05-24

**Authors:** Karin Verspoor, Judith Cohn, Cliff Joslyn, Sue Mniszewski, Andreas Rechtsteiner, Luis M Rocha, Tiago Simas

**Affiliations:** 1 Los Alamos National Laboratory, PO Box 1663, MS B256, Los Alamos, NM 87545, USA; 2 School of Informatics, Indiana University, 1900 East Tenth Street, Bloomington IN 47406, USA; 3 Cognitive Science Program, Sycamore Hall 0014, 1033 E. Third Street, Indiana University, Bloomington, IN 47405, USA

## Abstract

**Background:**

We participated in the BioCreAtIvE Task 2, which addressed the annotation of proteins into the Gene Ontology (GO) based on the text of a given document and the selection of evidence text from the document justifying that annotation. We approached the task utilizing several combinations of two distinct methods: an unsupervised algorithm for expanding words associated with GO nodes, and an annotation methodology which treats annotation as categorization of terms from a protein's document neighborhood into the GO.

**Results:**

The evaluation results indicate that the method for expanding words associated with GO nodes is quite powerful; we were able to successfully select appropriate evidence text for a given annotation in 38% of Task 2.1 queries by building on this method. The term categorization methodology achieved a precision of 16% for annotation within the correct extended family in Task 2.2, though we show through subsequent analysis that this can be improved with a different parameter setting. Our architecture proved not to be very successful on the evidence text component of the task, in the configuration used to generate the submitted results.

**Conclusion:**

The initial results show promise for both of the methods we explored, and we are planning to integrate the methods more closely to achieve better results overall.

## Background

We participated in the BioCreAtIvE evaluation (Critical Assessment of Information Extraction in Biology). We addressed Task 2, the problem of annotation of a protein with a node in the Gene Ontology (GO, ) [1] based on the text of a given document, and the selection of evidence text justifying the predicted annotation. We approached the task utilizing various combinations of two distinct methods. The first method is an unsupervised algorithm for expanding words associated with GO nodes. The second method approaches annotation as categorization of terms derived from the sentential neighborhoods of the given protein in the given document into nodes in the GO. This term categorization draws on lexical overlaps with the terms in GO node labels and terms additionally identified as related to those nodes. The system also incorporates Natural Language Processing (NLP) components such as a morphological normalizer, a named entity recognizer, and a statistical term frequency analyzer. The unsupervised method for expanding words associated with GO nodes is based on a probability measure that captures word proximity from co-occurrence data [2]. The categorization methodology uses our novel Gene Ontology Categorizer (GOC) technology [3] to select GO nodes which cover the terms in the input set, based on the structure of the GO.

BioCreAtIvE Task 2 had two subtasks for which we received evaluated results:

**Task 2.1 **– Given a <protein, document, GO node identifier> triple, return the evidence text from the document supporting the annotation of the protein to that GO node.

**Task 2.2 **– Given a <protein, document> pair, return annotations into the GO (in the form of GO node identifiers) for the given protein based on the given document, along with supporting evidence text from the document for each annotation. The number of annotations expected for the input pair, relative to each of the three branches of the GO (biological process, molecular function, and cellular component) was also provided.

## Methods

### Corpus pre-processing

Some pre-processing was performed on the document corpus. The original SGML documents were parsed to extract the Title, Abstract, and Body components, to normalize SGML character entities to corresponding ASCII characters (for instance, converting "&prime;" to an apostrophe), and to remove all formatting tags apart from paragraph markers.

### Morphological normalization

We morphologically normalized the documents using a tool we developed, called *BioMorpher*. BioMorpher is a morphological analysis tool built on the Morph tool originally developed at the University of Sheffield by Kevin Humphreys and Hamish Cunningham for general English. The Morph tool was extended to include large exception lists for biological text as well as to handle some morphological patterns not handled by the original tool.

### Term frequency analysis

As a pre-processing step, we performed a frequency analysis on the morphologically normalized documents to establish baseline frequencies for terms in documents throughout the corpus. In the dynamic processing of an input document, we selected representative terms for the document using a TFIDF filter (term frequency inverse document frequency, [4]). The TFIDF metric can be thought of as providing a measurement of the salience of a term in the document, relative to its general importance in the corpus. An extremely common domain term such as "protein" would have a low TFIDF score despite its prevalence in a particular document, while we would expect a term such as "necrosis" occurring in a document to have a higher TFIDF score since it is a term which will only appear in a small subset of the documents in the corpus.

### Protein recognition and context term selection

Swiss-Prot and TrEMBL identifiers were provided as input identifiers for the protein, so we needed to establish a set of names by which that protein (indicated by a Swiss-Prot identifier) could be referenced in the text. We made use of both the gene name and protein names that are in Swiss-Prot itself, when available, and a proprietary collection of protein name synonyms constructed by Procter & Gamble Company. The fallback case was to use the name filled in from the EBI TrEMBL human data. A script was applied to the TrEMBL names that generated variants of strings containing mismatched punctuation and parentheticals such as "(precursor)" or "(fragment)" which were felt not to be likely to occur directly in the text. The resulting database tables were used to construct a list which was dynamically loaded from the database into a GATE (General Architecture for Text Engineering, [5]) gazetteer processing module. This is a module which compiles the list of names into a finite state recognizer for the set of names, so that when a document is analyzed by the module each occurrence of a name in the list is identified in the document.

We chose this list-based strategy as it was straightforward to implement, and because protein reference identification was being addressed in BioCreAtIvE Task 1. The training data for Task 2 supported this strategy – a large majority (about 70%) of the training queries contained proteins that had names in our database.

The identification of occurrences of any known variant of a protein name facilitates identifying terms in the contextual neighborhood of the protein. Using another GATE module to identify sentence boundaries in combination with the gazetteer processor, we identify all sentences in the given document containing a reference to the protein given in the input query. This set of sentences is considered to be the contextual neighborhood of the protein, and all (morphologically normalized) terms are extracted from these sentences to establish a set of document-derived context terms for the protein. These terms are in turn associated with TFIDF weights calculated for each term in the document, and filtered to select the highest-ranked terms according to these weights.

### Unsupervised methodology for expanding words associated with GO nodes

Each node in the Gene Ontology (GO) is associated with a textual label, in addition to its numeric identifier. This label is intended to capture the meaning of the node, i.e. to reflect the underlying concept that the node represents. However, these labels tend to be relatively short (e.g. "membrane fusion" or "regulation of viral life cycle") and often the terms in a given label occur in many other labels (in particular terms such as "regulator/regulation" and "activity") throughout the GO. As such, the occurrence of an individual term that is part of a GO node label in a document may not be a sufficiently reliable indicator of the relevance of that GO node to the document. To address this, we utilized a method for expanding the set of terms associated with a given GO node. This method is based on the idea that the presence of words that are strongly associated with a GO node label are good indicators of that GO node, in addition to the terms that occur in the node label itself.

The <protein, document, GO node identifier> triples provided for training purposes, as well as those given as queries for Task 2.1, were used to determine sets of words related to GO nodes following a methodology developed for the *Active Recommendation Project *at Los Alamos [6]. After document pre-processing, we divided each document into paragraphs and calculated for each document a matrix of word occurrence in the paragraphs: *R*: *P *× *W*, where *P *is the set of all *m *paragraphs in a document, and *W *is the set of all *n *words. This is a Boolean matrix (*r*_*i*,*j *_∈ {0, 1}) that specifies if a given word occurred at least once in a given paragraph.

From the *R *matrices, we calculated a *word in paragraph proximity* matrix, *WPP*, for each document, using the co-occurrence probability measure below, as defined in [2]:



*WPP *denotes the association strength between pairs of words (*w*_*i*_, *w*_*j*_), based on how often they co-occur in the paragraphs of a given document. A value of *wpp *(*w*_*i*_, *w*_*j*_) = 0.3, means that words *w*_*i *_and *w*_*j *_co-occur in the same paragraphs 30% of the time that either one of them occurs. To avoid artificially high values of *WPP*, we computed this value only if the total number of paragraphs in which either of the words occurs (the denominator of the formula) is at least 3. Ideally, this value should be derived from the occurrence and co-occurrence distributions of words in a document's paragraphs, to prevent randomly co-occurring words from receiving high values of *WPP*. We did not compute such distributions for the BioCreative data, but rather used our results from other datasets used by the *Active Recommendation Project*, where, typically, a value of 3 dramatically reduces the chances of artificially high values of *WPP*.

**Figure 1:**
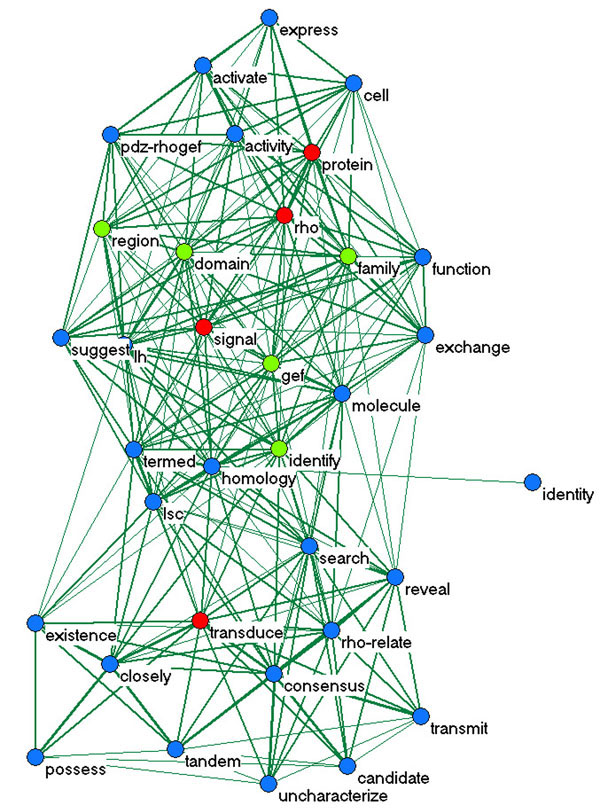
**Subnetwork of *WPP *with 34 words for document JBC_1999/bc005868. **The red nodes denote the words retrieved from the given GO annotation (0007266: "Rho", "protein", "signal", "transduce"): *W*_*GO*_. The blue nodes denote the words that co-occur very frequently (*wpp *> 0.5) with at least one of the red nodes: the co-occurrence neighborhood of the GO words. The green nodes denote the additional words discovered by our algorithm as described in the text. Only edges with *wpp *> 0.3 are shown.

We can think of *WPP *as an associative network of words. Indeed, the *WPP *matrix defines a fuzzy graph [7] where the vertices are words *w*_*i*_, and the edges are probability weights *wpp *(*w*_*i*_, *w*_*j*_). Such a graph can also be understood as an associative knowledge structure that represents how words co-occur in a given document, and therefore as an associative model of the knowledge stored in each document in terms of its constituent words [8]. As in any other co-occurrence method, the assumption is that words that frequently co-occur are associated with a common concept. Building a graph of co-occurrence proximity allows us to capture network associations rather than just pair-wise co-occurrence. Therefore, we expect concepts or themes (e.g. [9]) to be organized in more interconnected sub-graphs, or clusters of words. Figure 1 depicts a sub-graph of the *WPP *for one of the BioCreAtIvE documents (JBC_1999/bc005868).

**Figure 2:**
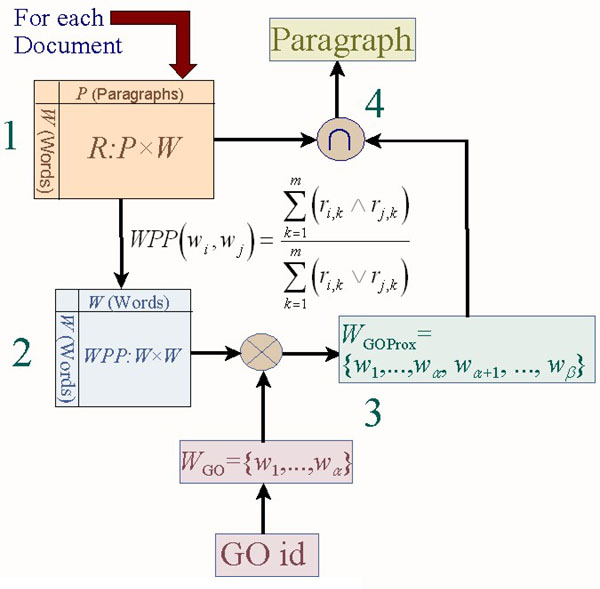
**GO node Word Expansion via proximity measure. **(1) For each document, a Boolean matrix of word occurrence in paragraphs (*R*) is created. (2) Co-occurrence proximity network *WPP *is computed. (3) Words in GO node label (*W*_*GO*_) are expanded (*W*_*GOProx*_) using *WPP*. (4) Intersection of vector of expanded GO node words (*W*_*GOProx*_) with word vectors for each paragraph in the document (columns of *R*) : paragraph with largest intersection is returned.

Next we set out to identify words associated with GO nodes. Using the GO nodes in the provided triples we retrieved the words from the GO node label. Let us refer to this set of words as *W*_*GO *_(the red nodes in Figure 1, for GO node 0007266). For each document, we then retrieved a set of words highly associated with the words in *W*_*GO *_in the relevant *WPP *network. Specifically, we returned the top 5 to 10 additional words with largest average value of *WPP *to all the words in *W*_*GO *_(the green nodes in Figure 1). The additional words thus discovered were used to expand *W*_*GO*_. Let us refer to the expanded set of words as *W*_*GOProx*_; the additional words are not found in the respective GO node label, but co-occur highly in a given document with the words in the GO node label. Given our assumption above, we can say that if a given GO node is about a specific concept or theme, then we expect the words in its label to co-occur with other words in any given document which also refer to this concept. Thus, the portion of text most appropriate as evidence text for the GO node is the portion where we find most of the words in the GO label plus the words that co-occur with those in the document. This process is depicted in Figure 2.

Run 1 submitted for Task 2.1 yielded a comparatively very good result (see Results presented below). In this run, for each <protein, document, GO node identifier> triple, we recommend a paragraph as evidence text for the respective GO node -- without ever using the protein identifier provided in the triplet. The recommended paragraph is selected by comparing the *W*_*GOProx, *_with each column of the document's word occurrence per  paragraph matrix *R.* The comparison was implemented by a vector intersection operation (step 4 in Figure 2). The columns of *R *are vectors of words occurring in a paragraph. We choose as evidence text for the GO node the paragraphs associated with the columns of *R *that yield the largest intersection with *W*_*GOProx*_. That is, paragraphs containing the largest number of words also found in *W*_*GOProx *_are selected.

### The Gene ontology categorizer

For Task 2.2, we were required to predict the appropriate GO node(s) associated with a protein based on the information in a given document. The methodology depicted in Figure 2, based on word proximities, cannot be used for this prediction as it depends on having the GO node label relevant for the query as an input. We therefore decided to pursue a strategy in which lexical overlaps between terms in the document and terms in the set of GO node labels were used to identify relevant GO nodes.

**Figure 3:**
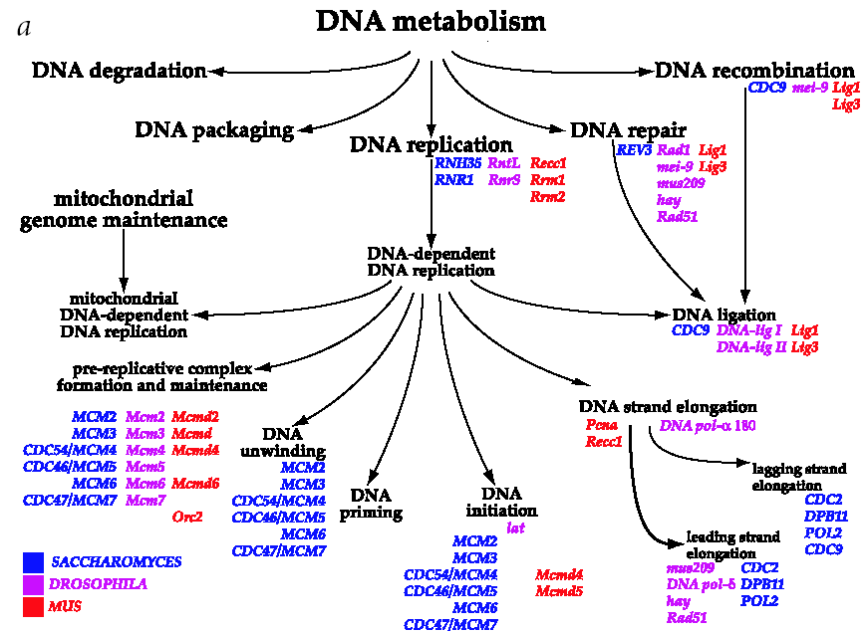
**A portion of the Molecular Function branch of the Gene Ontology. **Reprinted with permission from Nature from [1].

The GO, however, has a hierarchical structure such that evidence for the relevance of a particular GO node is also evidence for the relevance of its parent node. This is illustrated in the small portion of the GO shown in Figure 3 (reprinted with permission from [1]), where GO nodes, as functional categories, are shown in black, and the gene products annotated to those nodes are shown in color for the different model organisms. So, for example, evidence for "DNA ligation" is also evidence for "DNA repair", since "DNA ligation" is recorded as a child of "DNA repair". Thus DNA ligation is a specific kind of DNA repair.

In order to take the structure of the GO into consideration in this analysis, we employed a technology called the Gene Ontology Categorizer (GOC, [3,10]). GOC was originally developed to address what we call the *categorization *task in the GO: given a set of gene products, and annotations of those gene products to nodes in the GO, where in the GO do those genes appear? Are they all localized together in the structure, or in multiple groups, or spread out over a wide area? This problem had not actually been well defined or addressed previously, and presents novel problems for computer science (see Appendix A below).

In the original GOC algorithm, a set of gene products acts as a query. After identifying the set of nodes which are annotated to that set, GOC traverses the structure of the GO, percolating hits upwards, and calculating scores for each GO node. GOC then returns a rank-ordered list of GO nodes representing cluster heads. In the end, this provides an assessment of which nodes best cover the genes.

Note that we are *not *using "cluster" here in the sense of traditional clustering, e.g. *k*-means, but rather to indicate a set of nodes that are spatially close based on the structure of the ontology. A brief technical description and toy example of the base GOC's operation is provided in Appendix A, and see elsewhere [3].

Since GOC utilizes the structure of the GO to find the best nodes to cover or categorize a given set of input nodes, it was natural to extend it to address the question here, which is given a set of *terms*, where do *they *appear in the GO. Thus for BioCreAtIvE Task 2, GOC was extended in a number of ways: first to accept weighted query items, then to take terms as query items, and finally to provide data on which of the input terms contributed to the selection of each cluster head. Appendix A also includes technical information on these extensions.

Input terms are mapped to GO nodes via one of three mechanisms:

• **Direct**: The term occurs in the node label of GO node

• **Definitional**: The term occurs in the definition text associated with GO node

• **Proximity**: The term is one of the *W*_*GOProx *_terms related to a GO node through the proximity-based word expansion described above [2]

Direct and indirect associations are counted as distinct "hits" on a node and can be weighted differently.

GOC is run on the derived query consisting of the set of GO nodes which the input terms map to, and its output of ranked cluster heads is treated as an annotation of the original input protein, which can be directly compared to the correct answers provided by the organizers (see discussion below).

### Evidence text selection

We make use of two mechanisms for evidence text selection. The first is a simple sentence selection algorithm aimed at selecting one sentence out of the set of sentences containing a relevant protein reference to serve as the evidence text. The sentence selected is the sentence with the maximal intersection of terms in the sentence and terms reported by GOC to be used in the selection of the relevant cluster head/GO node (which in turn is a subset of the full set of context neighborhood input terms submitted to GOC).

The second algorithm, referred to below as the paragraph selection algortihm, draws on proximity measurement. In this case, we again consider the terms reported by GOC to be used in the selection of the relevant GO node. We evaluate the proximity of those terms to individual paragraphs in the document, using the document matrix R. The closest match using the vector intersection operation (Figure 2, step 4) is selected as the evidence.

### System operation

**Figure 4:**
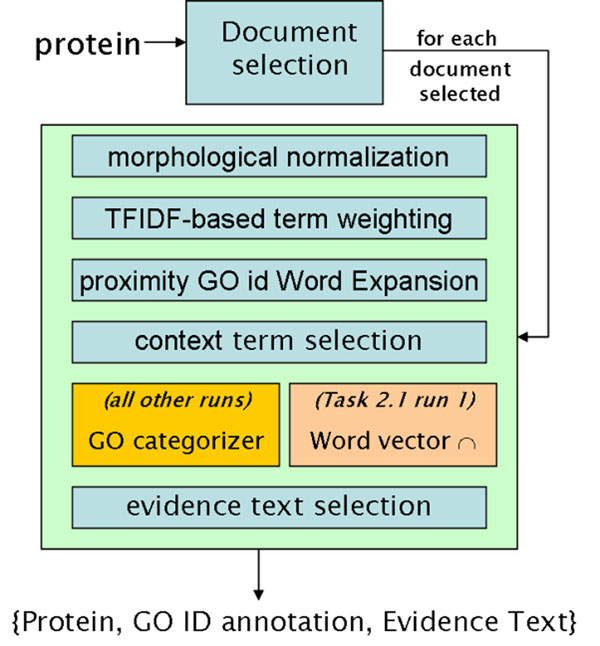
The Los Alamos System Architecture for BioCreAtIvE Task 2.

The architecture of the complete system is shown in Figure 4. For BioCreAtIvE tasks 2.1 and 2.2, the document selection portion is not relevant, as the documents were manually selected by the evaluators and provided in the input queries. There was an additional task, 2.3, which addressed selection of documents relevant to the annotation of a given protein. However, this task was not rigorously evaluated in BioCreAtIve, and so we do not report here on this component of the system.

As mentioned previously, morphological normalization, TFIDF-based term weighting, and proximity-based GO node word expansion are performed during preprocessing for each document. When executing a given query (for most runs, as we will outline below), we also perform context term selection in order to focus on terms that are most likely to be directly relevant to annotating the protein. These sets of terms together, with each term weighted by TFIDF to represent its significance, form the input items for subsequent processing.

We employ the GOC term categorization method to predict GO annotations (up to the provided limit of *n *annotations in a specific branch of the GO). The GOC output is then further used to select the evidence text for the GO assignment associated with each GO node annotation (cluster head), as described in the previous section.

We submitted 3 runs for each of tasks 2.1 and 2.2 (as well as a run for task 2.3 which was not scored). The runs consisted of the following configurations of the system:

#### Task 2.1

**Run 1**: A configuration bypassing GOC, utilizing only the GO label Word Expansion, based on proximity networks, followed by vector intersection of the columns of *R *and the expanded set of words associated with a GO node identifier, *W*_*GOProx*_, to discover paragraphs (essentially, the architecture of Figure 2).

**Run 2**: A configuration using the full system architecture including GOC, in which GOC is constrained to search for cluster heads only below the annotation given in the input query. Evidence selection consisted of the simple sentence selection algorithm.

**Run 3**: Same configuration as above for annotation portion. Evidence selection used the paragraph selection algorithm based on GOC results.

### Task 2.2

**Run 1**: A configuration using the full system architecture. Evidence selection consisting of the simple sentence selection algorithm.

**Run 2**: A configuration using the full standard system architecture. Evidence selection consisting of the paragraph selection algorithm based on GOC results.

**Run 3**: A configuration using the full system architecture, minus the sentence-based context term selection component, using instead the "fallback" scenario of selecting the top TFIDF-ranked terms in the document as a whole as the context terms for the protein. Evidence selection consisted of the paragraph selection algorithm based on GOC results.

## Results

Results were evaluated by professional annotators from the European Bioinformatics Institute (EBI) by considering the evidence text according to two criteria – whether the evidence text included a reference to the correct protein, and whether the evidence text directly referenced the GO node returned as the annotation. On each of these two dimensions, the text was evaluated as "high" (correct), "generally" (generally correct, perhaps referencing the correct family of proteins rather than the protein itself, or the parent of the target GO annotation rather than the target annotation itself), or "low" (incorrect). Overall, the evidence text was judged as "perfect" if it scored "high" on both of the criteria, and as "generally" when the protein was correct but the GO reference was "generally". The GO annotations were not evaluated independently from the evidence text in the official evaluation results.

The results for the two tasks are shown in Tables 1 and 2. We were user 7. On Task 2.1, run 1, we achieved a score of either perfect or generally good for 413 of the results; this corresponds to a good result for 38% of the 1076 queries. Focusing just on perfect results, our result was 263 (24%). In this configuration, we ignored the protein altogether and focused on the GO node-paragraph relationship. Nonetheless, we received a score of "high" on the protein mention measurement for 638 of the 1050 (61%) answers we submitted. This result reflects a high coherence between GO nodes and given proteins in the given documents, at least at the level of paragraphs.

Our results for the other runs we submitted for Task 2.1 were less good, achieving a perfect or generally good score for 83/86 (runs 2/3, respectively) of the queries, or about 8%.

Our Task 2.2 results were in general not good, as shown in Table 2 (user 7). However, it was discovered after the initial evaluation results were returned that there had been a problem with the evaluation of our submissions, as well as the submissions of user 17. We were allowed to select one run for reevaluation by the EBI annotators; we selected run 2. Table 2 shows the results after re-evaluation; approximately 5% "perfect" and 2% "generally" correct. The numbers in brackets indicate the original evaluation results for those runs. It is clear that the re-evaluation resulted in significantly more positive results, so that we can assume that the reported numbers for the runs 1 and 3 are also lower than the actual (corrected) results would indicate. We are also aware of a number of issues which contributed to our poor results, and which we have since addressed in part, and discuss below.

## Discussion

There are several important general issues in the evaluation that impacted our performance.

### Unknown proteins

We discovered that the test data contained many protein IDs that were not yet available in SwissProt, in stark contrast to the training data. Only 58 of the 286 (20%) proteins referenced in Task 2 evaluation queries were named in our database; 29/138 (21%) of Task 2.1 proteins and 19/138 (14%) of Task 2.2 proteins. With respect to queries, only 153/1076 (14%) of Task 2.1 queries and 44/435 (10%) of Task 2.2 queries included proteins for which we had names. We were able to fall back to the names in the TrEMBL database, but these are of poor quality and usually there is only one name, not a full set of synonyms for a protein; often we did not find any occurrences of these names in the query document. This issue had a big impact on our ability to focus in on text within documents that was directly relevant to the protein of interest (see further discussion of this problem, below). On the other hand, post-hoc analysis of our (corrected) evaluation results for Task 2.2, run 2 showed that 16 of the 19 "perfect" and 8 of the 9 "generally" results actually were achieved for proteins *not *in our database. This suggests two possible problems. The first is that perhaps the names that we do have in our database are inadequate for effective protein reference identification and we should explore more shopisticated protein reference recognition techniques (such as those explored in BioCreAtIvE Task 1). The second potential explanation for these results is that the use of a single sentence as context for terms related to annotation of the protein of interest is too narrow. We should therefore experiment with the size of left and right context windows around protein references to achieve better results.

### Assessing annotation accuracy

The methodology followed by the evaluators of Task 2.2 focused on the evidence text selection, measuring whether the selected evidence text for a given query mentioned both the protein of interest, and the function/process/component indicated by the target GO node. The prediction of the GO node itself was not evaluated independently from the evidence text returned as justification for the prediction.

Our interpretation of the task was that there were two results: prediction of the GO node and selection of the evidence text. While in some of the runs, our overall results were not strong, our independent investigations show that our overall performance is better when considering annotation (GO node prediction) distinctly from evidence text selection. We will show this in what follows.

Since completion of the formal BioCreAtIvE evaluation, we have refined, improved, and measured our annotation results in a number of ways. First, there is a free parameter *s *to GOC called the *specificity*, which represents the extent to which the user values results which are either "low" or "high" in the GO hierarchy (see the Appendix and elsewhere [3]). Succinctly, higher values of *s *will tend to give higher scores to nodes which are lower in the GO, and thus represent more specific or concrete concepts; lower values of *s *will tend to give higher scores to nodes which are higher in the GO, representing more general or abstract concepts.

In practice, GOC tends to converge (in different directions) for values of *s *less than 2 or greater than 7, neither of which produces optimal results. But because GOC is itself a novel technique, at the time of the results  submission we had not yet refined our sense of the use of this parameter, and hence set it to be much higher than appropriate (*s *= 7). We shall see that this was an improper choice, with stronger results for moderate levels of specificity.

For each query we were instructed to provide a certain number *n *of annotations, and after the fact we were told what those correct annotations were. GOC returns a rank-ordered list usually longer then *n*, and so we cut this list off at *n *nodes, even if a correct answer might have occurred lower down in the list. Thus we end up with two sets of *n *nodes from the GO – our *n *annotation predictions and the *n *correct annotations.

To calculate our annotation accuracy, we can check how many of our answers match the correct answer exactly, but this doesn't account for "near misses", where we might return a parent, child, or sibling of the correct answer, and still wish to count this as some kind of correct response. Ultimately, this problem becomes that of measuring the amount of overlap between two sets of GO nodes, which is actually a difficult mathematical problem, which we [3,11] and others (e.g. [12]) are addressing. A detailed treatment of this subject is beyond the scope of this paper, but for our purposes, we measured "near misses" between two nodes *p *and *q *using the following categories:

• **Direct hit: ***p *= *q*

• **Nuclear family: **a direct hit, or *p *is a child, parent, or sibling of *q*.

• **Extended family: **a nuclear family hit, or *p *is grandparent, grandchild, cousin (grandchild of a grandparent or grandparent of a grandchild), aunt/uncle (child of a grandparent), or a niece/nephew (grandchild of a parent), of *q*.

• **Ancestor: ***p *is any ancestor of *q*.

**Figure 5:**
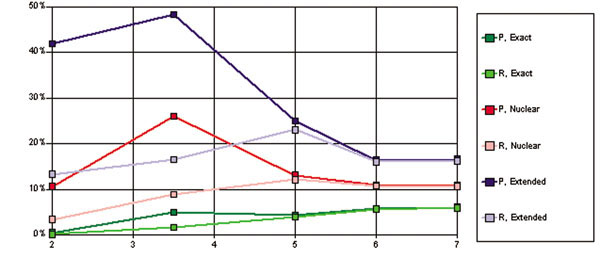
**Precision vs. Recall for different values of Specificity, *s *.**Paired precision (*P*) and recall (*R*) results as a function of specificity broken out by inclusive "family groups" as mentioned in the text. Note that recall is bounded above by precision, due to the need to cut off the number of GOC cluster heads considered based on the number of requested results. The x axis indicates the value of specificity (*s*); the y axis represents the value of precision or recall, as a percentage.

Precision and recall as a function of specificity *s *across these different categories are shown in Figure 5. Results are especially poor for direct hits and very high specificity. A high specificity (*s *= 7) was used for all of the GOC-based runs submitted. For Task 2.2, the submitted results were therefore not as good as they might have been, with 6% precision and 5.9% recall for direct hits, 10.8% precision and 10.5% recall within the correct nuclear family, and 16.6% precision and 16.2% recall within the correct extended family. For moderate levels of specificity at the level of nuclear and extended families, our results approach 50% precision.

**Figure 6:**
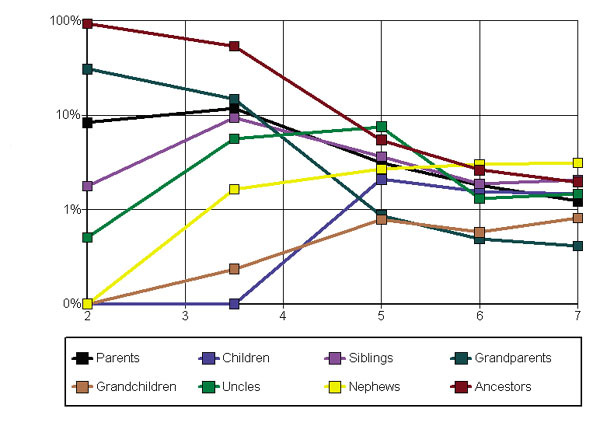
**Precision for different values of Specificity, *s *.**Log of precision as a function of *s*, broken out by the distinct (non-cumulative) family relations. By constrast, the precision results for "nuclear family" in Fig. 5 is the sum of direct hits from Fig. 5 and parents, children, and siblings from this figure.

Note that due to the list cutoff, recall is bounded above by precision. Thus Figure 6 shows a more detailed analysis for precision only, and furthermore breaks out the family groups by their individual constituents (e.g. parents and siblings). Results are shown on a log scale.

Some of the results appear impressive, for example approaching 100% for all ancestors and low specificity. This is misleading, since simply the topmost GO nodes like "biological process" and "gene ontology" are identified. However, looking at moderately "tight" neighborhoods like parents and grandparents, in family groups like nuclear and extended, reveals a moderately successful approach to automated functional annotation into the GO.

### Discussion, GOC-based runs

Due to the "unknown proteins" problem described above, the protein neighborhood terms input to GOC were in most instances the top TFIDF-ranked terms for the document as a whole, rather than coming from a coherent textual neighborhood around the protein. This had several implications. First, GOC may have been "overseeded" – since the input terms were derived from across the document, they may have matched very dispersed nodes in the GO. This would make it difficult for the GOC algorithm to confidently select a covering node for the input terms. Second, evidence text selection on the basis of overlap with or proximity to terms from across the document is difficult; it is unlikely that any single sentence/paragraph matches more than a few of these terms.

The overseeding may have worsened the impact of an additional difficulty. The number of terms from the GOC input set used to rank a GO node was typically very small – normally 1–3 terms – and only this subset of terms was passed on to the two evidence selection algorithms. The motivation underlying this approach was to enable the evidence text selection for a GO annotation to proceed on the basis of only those document terms relevant to that annotation. In practice, given the small and weakly coherent sets of terms that were generated, this created great difficulty for reliably selecting a contiguous chunk of text focused on that GO node. This would have impacted the quality of the evidence text selected, and hence our overall evaluation results. This problem could likely have been ameliorated by incorporating the strategy from Task 2.1, Run 1, utilizing all available information about the selected GO node, rather than limiting ourselves to terms from the context window.

Finally, we would like to explore the interaction between TFIDF weights and the importance of a term in the GO. Preliminary analysis suggests that there are very frequent terms in the GO with relatively high TFIDF scores in the corpus; this would unfairly value those terms in GOC and exacerbate the overseeding problem. Some adjustment of the weighting scheme to better take into consideration the terminological structure of the GO is therefore warranted.

### Discussion, proximity network-based word expansion and evidence text selection

While the proximity network-based word expansion proved to be a very useful technique, giving us good results on Task 2.1, the evaluator comments indicated that they were often unhappy with paragraphs as the basic unit for evidence text. To address this, we envision several changes. We could apply the proximity measurements at the sentence level, rather than the word level; we could explore metrics for recognizing excessively long paragraphs and splitting them at positions of subtle topic change; or we could try to use more linguistic (structural) analysis to focus in on the core information expressed and narrow the text returned.

There are some additional ways to build on our results. We could calculate a global word proximity matrix, rather than one matrix per document, which should strengthen our confidence in the relationships between words, as well as relating any given word to more words due to consideration of its occurrence across the document corpus. We could also incorporate semi-metric analysis of the word proximities [2] to find additional (indirectly) related words, even if they do not directly co-occur in the corpus.

## Conclusion

There is still significant room for improvement on this task. This is evidence of the complexities of automatic annotation of GO nodes to proteins based on a single document, where complexities arise both from the structure of the GO itself and the difficulties of annotating into a large and extremely hierarchical structure, and from the ambiguous nature of text. However, the initial results show promise for both of the methods we explored, and further analysis has helped us to better understand the impact of the various parameters of the system. We are planning to integrate the two methods explored in this study more closely to achieve better results overall.

## Authors' contributions

KV managed the project, developed the text pre-processing and NLP tools, built the integrated infrastructure of the complete system, and identified the GOC extensions necessary for our solution. JC provided database support, specifically for protein name management. CJ participated in the design of the solution, defined the mathematical extensions to GOC, and analyzed the GO node annotation results. SM was responsible for the code development of GOC and the implementation of the extensions in the code. AR worked on BioCreAtIvE Task 2.3 which was not evaluated in the end. LMR participated in the design of the proximity analysis and paragraph selection algorithms. TS developed the code for TFIDF and proximity analysis, and implemented paragraph selection. KV, CJ, and LMR contributed to the writing of the manuscript.

## Appendix A: The gene ontology categorizer (GOC) and its extensions

GOC is an algorithm for categorization in hierarchies represented as partially ordered sets (posets [13]). Posets are distinguished from networks, which are represented as directed graphs: while every poset is a directed graph, the converse is not true. In particular, the GO is a collection of posets, two each (is-a and has-part) for each of the three branches Molecular Function, Cellular Component, and Biological Process.

Space precludes a full explication of GOC, which would furthermore be redundant with prior published work [3]. Therefore a synoptic account is provided here, focusing on the extensions to GOC for this task. For full details about the base GOC, see [3].

GOC begins by casting the nodes of the GO as a set *P *with a **partial order ≤ : **a reflexive, symmetric, and anti-transitive binary relation over the elements of *P*. Here ≤ is actually the union of all the is-a and has-part links, so that *p *≤ *q *if either *p *is a kind of *q *or *p *is a part of *q*. Together *P *and ≤ yield a structure called a **partially ordered set **(or **poset**) **P **= (*P*, ≤).

Two nodes *p*,*q *∈ *P *are called **comparable **when there is a unidirectional path, called a **chain **[13], in the GO between them, so that, either *p *is a kind of *q *or *p *is a part of *q*, with *p *≤ *q *; or *vice versa*, so that *q *≤ *p*. Note that many chains may connect two comparable nodes.

Then, features of GO nodes are cast as a set of **labels ***X*, and can be, for example, the gene products annotated to GO nodes, or in our case are the terms making up the labels of each GO node. An **annotation function ***F *: *X *→ 2^*P *^then assigns to each feature (term) *x *∈ *X *the collection of GO nodes *F*(*x*) ⊆ *P *with which they are associated. Altogether, we construct a mathematical structure called a **POSet Ontology (POSO) *O ***= (***P***,*X*, *F*).

Between all pairs of comparable nodes *p *≤ *q *we define a **pseudo-distance ***δ*(*p*, *q*) to indicate how "high" *q *is above *p*. While many pseudo-distances are possible, in practice we use four: the length of the minimum chain between them, denoted *δ*_*m*_; the length of the maximal chain *δ*_*x *_; the average of these *δ*_*ax *_= (*δ*_*m *_+ *δ*_*x*_)/2 ; and the average of the lengths of all the chains between *p *and *q *denoted *δ*_*ap*_.

We also use a **normalized pseudo-distances ** derived by dividing *δ *by the **height **of ***P***, which is the size of its largest chain. Effectively, an absolute pseudo-distance measures the number of "hops" between two comparable nodes *p *≤ *q*, while a normalized pseudo-distance measures what *proportion *of the height of the *whole *poset ***P ***is taken up between *p *and *q*.

**Figure 7:**
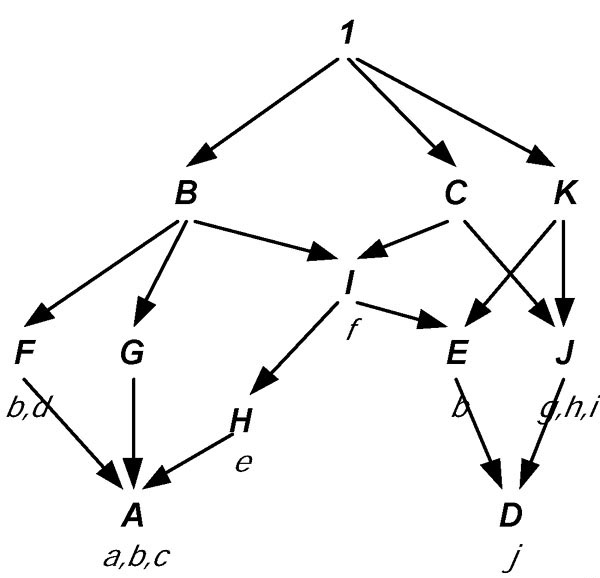
**A toy example of a labeled poset. **GO nodes are modeled by nodes with capital letters, with gene labels annotated to them in lower case. Note that the structure is neither a tree nor a lattice, but technically, the Hasse diagram of a poset **P**.

A toy example of a POSO is shown in Figure 7, where we have *P *= { *A*, *B*, ..., *K *}, *X *= { *a*, *b*, ..., *j *}, the partial order ≤ is as indicated in the figure, and e.g. *F*(*b*) = { *A*, *E*, *F *}. The height of ***P ***is 4, and *A *≤ *B *are comparable nodes connected by three chains *A *≤ *F *≤ *B*, *A *≤ *G *≤ *B*, and *A *≤ *H *≤ *I *≤ *B*, so that *δ*_*m *_(*A*, *B*) = 3, *δ*_*x *_(*A*, *B*) = 4, *δ*_*ax *_(*A*, *B*) = 2.5, *δ*_*ap *_(*A*, *B*) = 2.33, and e.g. (*A*, *B*) = 3/4.

Given a pseudo-distance and a set of labels of interest *Y *⊆ *X*, we then want to develop a **scoring function ***S*_*Y*_(*p*) which returns the score of a node *p *∈ *P *based on the other nodes in the GO which are annotated by the requested labels *Y*, and the poset structure of the GO. We have an **unnormalized score ***S*_*Y*_: *P *→ **R**^+ ^which returns an "absolute" number greater than or equal to zero, and a **normalized score ** : *P *→ [0,1] which returns a number, between 0 and 1, indicating the score relative to a theoretical maximal value. We also allow the user to choose the relative value placed on coverage vs. specificity by introducing a parameter *s *∈ {...,-1,0,1,2,3...}, where low *s *emphasizes nodes higher in the GO which are more likely to cover a label of interest, and high *s *emphasizes nodes lower in the GO which are more likely to be very specific annotations to a label of interest.

Since both the normalized and unnormalized scoring function can use either the normalized or unnormalized distances, there are four possible scoring functions used in the original GOC [3], letting *r *= 2^*s*^, and thereby incorporating specificity as shown in table 4.

Output for the example in Fig 4 is shown in Table 3 for the query *Y *= { *c*, *e*, *i *}, specificity values *s *= *-1*, *1*, and *3*, the "doubly-normalized" score , and the normalized pseudo-distance . In addition to scoring each node, GOC identifies cluster heads, which are shown in bold; and so-called "secondary cluster heads" which are cluster heads which are ancestors of a primary cluster head, and which are labelled with *.

For the BioCreAtIvE Task 2 the following changes were made to the base GOC algorithm described above:

• Label sets *X *were allowed to be terms as well as gene products.

• Queries took the form of lists of terms weighted as described above.

• Since each item of the list "hits" a collection of GO nodes with its particular weight, the query as a whole implicates a collection of GO nodes in a complex way. When the weights are carried over from the query terms to the list of nodes, the structure which results is called a **fuzzy bag **of *P*, denoted here *Q *⊲ *P*.

So the fuzzy bag *Q *is an unordered collection of possibly duplicated nodes *p *∈ *P *equipped with weights *w *: *Q *→ [0,1]. As an example, a query could be

{ ("protein biosynthesis", 0.8), ("biosynthesis", 0.8), ("lipoprotein", 0.7) }

resulting in the fuzzy bag of nodes

*Q *= { (GO:0042157: lipoprotein metabolism, 0.7),

(GO:0006412: protein biosynthesis, 0.8),

(GO:0006412: protein biosynthesis, 0.8),

(GO:0042158: lipoprotein biosynthesis, 0.8),

(GO:0042158: lipoprotein biosynthesis, 0.7) }.

Note the duplicate items in the bag, in particular the node GO:0006412 is present twice with weight 0.8, receiving one contribution from the query term ("protein biosynthesis", 0.8) and another from the query term ("biosynthesis", 0.8).

The original scoring functions above are then modified as shown in Table 5, again letting *r *= 2^*s*^.

where *|Q| *is the size of the query, taken as the cardinality of the bag *Q*: 

## Figures and Tables

**Table 1:** Results across all users for BioCreAtIvE Task 2.1.

**User**, **Run**	**# results**	**"perfect"**	**"generally"**
4, 1	1048	268 (25.57%)	74 (7.06%)
5, 1	1053	166 (15.76%)	77 (7.31%)
5, 2	1050	166 (15.81%)	90 (8.57%)
5, 3	1050	154 (14.67%)	86 (8.19%)
7, 1	1050	263 (25.05%)	150 (14.29%)
7, 2	1856	43 (2.32%)	40 (2.16%)
7, 3	1698	59 (3.47%)	27 (1.59%)
9, 1	251	125 (49.80%)	13 (5.18%)
9, 2	70	33 (47.14%)	5 (7.14%)
9, 3	89	41 (46.07%)	7 (7.87%)
10, 1	45	36 (80.00%)	3 (6.67%)
10, 2	59	45 (76.27%)	2 (3.39%)
10, 3	64	50 (78.12%)	4 (6.25%)
14, 1	1050	303 (28.86%)	69 (6.57%)
15, 1	524	59 (11.26%)	28 (5.34%)
15, 2	998	125 (12.53%)	69 (6.91%)
17, 1	412	0 (0.00%)	1 (0.24%)
17, 2	458	1 (0.22%)	0 (0.00%)
20, 1	1048	300 (28.63%)	57 (5.44%)
20, 2	1050	280 (26.72%)	60 (5.73%)
20, 3	1050	239 (22.76%)	59 (5.62%)

Evaluation results on the evidence text selected for Task 2.1. A "perfect" evaluation indicates that the evidence text refers to both the correct protein and the correct GO node. A "generally" evaluation indicates that it refers to the correct protein and that the reference to a GO node is somewhat too general. The Los Alamos team is user 7.

**Table 2:** Results across all users for BioCreAtIvE Task 2.2.

**User**, **Run**	**# results**	**"perfect"**	**"generally"**
4, 1	661	78 (11.80%)	49 (7.41%)
7, 1	153	1 (0.65%)	1 (0.65%)
7, 2	384	19 (4.95%) [1]	9 (2.34%) [1]
7, 3	263	2 (0.76%)	10 (3.80%)
9, 1	28	9 (32.14%)	3 (10.71%)
9, 2	41	14 (34.15%)	1 (2.44%)
9, 3	41	14 (34.15%)	1 (2.44%)
10, 1	120	35 (29.17%)	8 (6.67%)
10, 2	86	24 (27.91%)	6 (6.98%)
10, 3	116	37 (31.90%)	11 (9.48%)
15, 1	502	3 (0.60%)	8 (1.59%)
15, 2	485	16 (3.30%)	26 (5.36%)
17, 1	247	52 (21.05%) [1]	23 (9.31%) [0]
17, 2	55	1 (1.82%)	0 (0.00%)
17, 3	99	1 (1.01%)	1 (1.01%)
20, 1	673	20 (2.97%)	30 (4.46%)
20, 2	672	38 (5.65%)	26 (3.87%)
20, 3	673	58 (8.62%)	27 (4.01%)

Evaluation results on the evidence text selected for Task 2.2. See legend for Table 1. In this task, evaluation of the GO node reference was done with respect to the predicted GO annotation provided by the system.

**Table 3:** Original GOC output in the toy example. GOC output for values of specificity *s *∈ { -1,1,3}.

	*s *= *-1*		*s *= *1*		*s *= *3*	
Rank		*p*		*p*		*p*

1	0.7672	***C***	0.5467	***H***	0.3893	***H***
2	0.6798	1*	0.3867	C*	0.3333	*A;* ***J***
3	0.6315	*H*	0.3333	*A;I;J*		
4	0.5563	*I*			0.0617	C*
5	0.5164	*B*			0.0615	*I*
6	0.3333	*A;J*	0.2400	B*	0.0559	*F;G;K*
7			0.2267	1*		
8	0.2981	*F;G;K*	0.2133	*F;G;K*		
9					0.0112	*B*
10					0.0060	***1***

**Table 4:** Original GOC scoring functions, letting r=2*^s^*

	Score	
	
Distance	Unnormalized	Normalized
Unnormalized		
Normalized		

**Table 5:** Modified GOC scoring functions for fuzzy bags, letting r=2*^s^*

	Score	
	
Distance	Unnormalized	Normalized
Unnormalized		
Normalized		
